# Pulmonary function and endothelial damage in patients undergoing renal replacement therapy: a cross-sectional study

**DOI:** 10.1590/2175-8239-JBN-2025-0222en

**Published:** 2026-08-24

**Authors:** Italo Caldas Silva, Gdayllon Cavalcante Meneses, Alice Maria Costa Martins, Alexandre Braga Libório, Elizabeth De Francesco Daher, Ronaldo de Matos Esmeraldo, Nataly Gurgel Campos, Tainá Veras de Sandes-Freitas

**Affiliations:** 1 Universidade Federal do Ceará, Programa de Pós-graduação em Ciências Médicas, Fortaleza, CE, Brazil.; 2 Universidade Federal do Ceará, Faculdade de Farmácia, Odontologia e Enfermagem, Fortaleza, CE, Brazil.; 3 Universidade de Fortaleza, Programa de Pós-graduação em Ciências Médicas, Fortaleza, CE, Brazil.; 4 Hospital Geral de Fortaleza, Setor de Transplante Renal, Fortaleza, Brazil.; 5 Universidade Federal do Ceará, Programa de Pós-graduação em Fisioterapia e Funcionalidade, Fortaleza, CE, Brazil.

**Keywords:** Kidney Transplantation, Renal Insufficiency, Chronic, Respiratory System, Respiratory Function Tests, Biomarkers

## Abstract

**Objective:**

To compare pulmonary function and biomarkers of endothelial injury between hemodialysis patients with end-stage renal disease (ESRD) and kidney transplant (KT) recipients.

**Methods:**

Cross-sectional study including 23 patients on dialysis for ≥ 24 months and 23 patients transplanted for ≥ 12 months, with a glomerular filtration rate ≥ 40 mL/min/1.73 m^2^, matched by sex and age. Pulmonary function was analyzed by maximal inspiratory and expiratory pressure (MIP and MEP), forced vital capacity (FVC), forced expiratory volume in one second (FEV1), and the Tiffeneau index. Endothelial damage was assessed using syndecan-1, intercellular adhesion molecule-1 (ICAM-1), vascular cell adhesion molecule (VCAM-1), and angiopoietin-2 (Ang-2).

**Results:**

Both groups had poor performance in pulmonary function tests. The percentage of patients reaching the predicted MIP, MEP, FEV1, and FVC values was low and similar between groups (43.5%, 4.3%, 0%, and 17.4%, respectively). There were no differences in the observed/predicted ratios for MEP (66 ± 17%), FEV1 (60 ± 18%), and FVC (76 ± 22%), or in the Tiffeneau index (0.8 [IQR 0.6–0.9]). KT patients showed lower MIP percentages (82 ± 19 vs. 94 ± 12%; p = 0.019). In the KT group, endothelial damage was significantly inversely correlated with pulmonary function parameters, and this group had lower levels of VCAM-1 (1,589 [IQR 1,009–1827] vs 2,302 [IQR 1,642–3,540] ng/mL; p = 0.001), Ang-2 (0.17 [IQR 0.01–1.14] vs 0.75 [IQR 0.30–1.29] ng/mL; p = 0.040), and syndecan-1 (47.9 [IQR 33.1–67.8] vs 195.8 [IQR 126.9–286.8] ng/mL; p < 0.001).

**Conclusion:**

Despite better endothelial function, KT was not associated with superior pulmonary function, suggesting a multifactorial pathophysiology for lung impairment.

## INTRODUCTION

Chronic kidney disease (CKD) patients may experience pulmonary dysfunction secondary to anemia, hypervolemia, pulmonary congestion, and, mainly, to respiratory muscle impairment caused by disuse atrophy, protein imbalance, uremic myopathy, chronic inflammation, oxidative stress, and endothelial damage^1–4^. Kidney transplantation (KT) potentially restores kidney function and, consequently, most of these disorders, improving long-term patient survival and quality of life^5,6,7,8^.

Previous studies have reported a partial improvement in pulmonary function after KT, attributing this recovery to the reversal of the uremic environment^9,10^. Beyond uremia, evidence indicates that KT improves endothelial dysfunction and reduces systemic inflammation, which may also contribute to the partial reversal of pulmonary dysfunction^11,12^.

It is intuitive to assume that recovery of renal function after KT results in full restoration of pulmonary function. However, there is a paucity of evidence on this topic. This novel study aims to compare patients with end-stage renal disease (ESRD) on dialysis with stable KT recipients. To further explore the mechanisms underlying respiratory impairment, we evaluated biomarkers of endothelial dysfunction in both groups.

## METHODS

Cross-sectional study including patients with ESRD on hemodialysis at a single dialysis center and KT recipients from a single transplant center, matched 1:1 by age and sex.

Patients undergoing hemodialysis for more than 24 months were selected non-probabilistically for the dialysis group. For the KT group, we enrolled KT recipients with ≥ 12 months of follow-up, aged between 18 and 70 years, with an estimated glomerular filtration rate (eGFR) ≥ 40 mL/min/1.73 m^2^, calculated using the CKD-EPI equation, and who were able to understand and perform the pulmonary evaluation procedures. Patients with a history of chronic obstructive pulmonary disease, asthma, acute myocardial infarction in the past three months, decompensated heart disease, or active infectious process were excluded from both groups. Those who had participated in any study involving physical exercise for less than six months or who were athletes or regular exercisers (more than twice a week) were also excluded.

To estimate the sample size, the following were considered: a) a significance level of 5%; b) a statistical power of 90%; c) forced expiratory volume in one second (FEV1) and forced vital capacity (FVC) as the outcome variables–using an expected difference of 0.86 and a standard deviation of 0.7 for FEV1, and an expected difference of 0.78 and a standard deviation of 0.7 for FVC, based on the literature^12,13^; and d) 20–30% of estimated withdrawals, missing data, and losses to follow-up.

The study was conducted in accordance with the ethical standards of Resolution No. 466/2012 of the Brazilian National Health Council and the Declaration of Helsinki and was approved by the Research Ethics Committee (CEP) of the *Hospital Geral de Fortaleza* (approval No. 2,794,399). Each patient included in the study provided written informed consent. Individual records and information were deidentified and anonymized prior to analysis.

Regarding the study procedures, the following clinical and demographic data were analyzed: sex, age, weight, height, body mass index (BMI), history of diabetes, hypertension, previous or current smoking, causes of ESKD, time on hemodialysis, and time after KT.

The analyzed pulmonary function parameters were maximal expiratory pressure (MEP), maximal inspiratory pressure (MIP), FEV1, FVC, and the Tiffeneau index, assessed before dialysis sessions in the dialysis group. MIP and MEP measurements were performed using an MR® manovacuometer. After three measurements, the highest value was considered for analysis. A portable ONE FLOW RANGE spirometer (Clement Clarke International) was used to assess spirometric parameters (FEV1 and FVC), according to the criteria established by the American Thoracic Society (1995).

The predicted MIP and MEP values were estimated using the following formulas^14^:

- Predicted MIP = 149.33 - 1.14 × age, if male; and 74.25 - 0.46 × age, if female.
- Predicted MEP = 183.31 - 1.26 × age, if male; and 119.35 - 0.68 × age, if female.

The predicted FEV1 and FVC values were estimated using the following formulas^12^:

- Predicted FEV1 = (height × 0.0473) - (age × 0.0281) - 3.145, if male; and (height × 0.0338) - (age × 0.0210) - 1.782, if female.
- Predicted FVC = 4.569 - (height × 0.0590) - (age × 0.0281), if male; and 2.967 - (height × 0.0433) - (age × 0.0164), if female.

The endothelial biomarkers were quantified from the isolated serum samples, using human ELISA kits.

ELISA assays were performed according to the manufacturer’s instructions for use. Syndecan-1: kit ab46506 (Abcam^®^); intercellular adhesion molecule-1 (ICAM-1), kit ab47349 (Abcam^®^); vascular cell adhesion molecule (VCAM-1): kit ab47355 (Abcam^®^); angiopoietin-2 (Ang-2): kit DY623 (R&D Systems^®^). Blood samples for biomarker analysis were collected 10–30 minutes before the hemodialysis session in the dialysis group. In the KT group, blood samples were collected before pulmonary function testing. Samples were collected in appropriate serum separator tubes, and the serum was aliquoted and stored appropriately at -80°C until analysis.

Statistical analysis was performed. Continuous variables with a normal distribution were summarized as means ± standard deviations and compared using Student’s t-test. Variables with a non-normal distribution were expressed as medians and interquartile ranges (IQR) and compared using the Mann-Whitney test. The Shapiro-Wilk test was used to assess normal distribution. Categorical variables were summarized as frequencies and proportions and compared using Fisher’s exact or chi-square (*χ* ^2^) tests. Spearman’s correlation coefficient (rho) was used to assess correlations between normally and non-normally distributed numerical variables. Data were analyzed using the Statistical Package for Social Sciences (SPSS) version 23.0 for Macintosh (IBM, Armonk, NY, USA). Vertical scatterplots were created using GraphPad Prism 5 for Windows (version 5.01). p < 0.05 was considered statistically significant.

## RESULTS

A total of 46 patients were included in the study, 23 in each group. Despite the groups being comparable, patients in the dialysis group were older than those in the KT group (51.1 ± 5.7 vs. 46.0 ± 5.0 years; p = 0.002). The groups differed regarding CKD etiology and history of diabetes, with a higher proportion of diabetic patients among those on dialysis (47.9% vs. 4.3%; p = 0.001). Table 1 presents the demographic data.

**Table 1.** Baseline demographic and clinical characteristics of dialysis patients and kidney transplant recipients.

	Total (N = 46)	Dialysis group (N = 23)	KT group (N = 23)	p-value
Gender – male, n (%)	23 (50)	12 (52.2)	11 (47.9)	0.774
Age (years), mean ± SD	49.5 ± 5.9	51.1 ± 5.7	46.0 ± 5.0	0.002
BMI (kg/m^2^), mean ± SD	25.6 ± 3.1	26.5 ± 3.5	24.7 ± 2.5	0.058
Time on dialysis (months), median (IQR)	36.0 (21.8–49.5)	36.0 (21.5–48.0)	36.0 (24.0–54.0)	0.851
Time after KT (months), median (IQR)	24.0 (17.5–48.0)	NA	24 (17.5–48.0)	NA
CKD etiology, n (%)				0.001
CGN	16 (34.8)	8 (34.8)	8 (34.8)	
Hypertension	10 (21.7)	7 (30.4)	3 (13.0)	
Unknown	10 (21.7)	0 (0)	10 (43.5)	
Diabetes	9 (19.5)	8 (34.8)	1 (8.7)	
PKD	1 (2.2)	0 (0)	1 (4.3)	
Comorbidities, n (%)				
Hypertension	28 (60.9)	16 (69.6)	12 (52.2)	0.232
Diabetes	12 (26.1)	11 (47.9)	1 (4.3)	0.001
Current or former smoking, n (%)	8 (17.4)	6 (26.1)	2 (8.7)	0.124
eGFR (mL/min/1.73 m^2^), mean ± SD	75.2 ± 27.6	NA	75.2 ± 27.6	NA
Maintenance IS regimen, n (%)				NA
TAC-mTORi	15 (36.6)	NA	15 (65.2)	
TAC-MPA	9 (19.6)	NA	9 (39.1)	
ST-based IS regimen, n (%)	9 (19.6)	NA	9 (39.1)	NA

Abbreviations – KT: kidney transplant; BMI: body mass index; CKD: chronic kidney disease; CGN: chronic glomerulonephritis; PKD: polycystic kidney disease; eGFR: estimated glomerular filtration rate; IS: immunosuppression; TAC: tacrolimus; mTORi: mTOR inhibitor; MPA: mycophenolate; ST: steroids.

Regarding pulmonary function parameters, there were no differences between the groups in observed MIP or in the percentage of patients who achieved the predicted MIP. Paradoxically, the observed/predicted MIP ratio was lower among transplant recipients (82 ± 19% vs. 94 ± 12%; p = 0.019). In the KT and dialysis groups, observed MIP values were not significantly lower than the predicted values. Regarding MEP, transplant patients had higher observed values (70 [IQR 60–100] vs. 60 [IQR 50–70] cm/H^2^O; p = 0.020), although no significant differences were observed in the percentage of patients who achieved the predicted MEP or in the observed/predicted MEP ratio. Both groups had MEP values lower than those predicted for age, height, and sex (Table 2).

**Table 2.** Pulmonary function in dialysis patients and kidney transplant recipients.

	Total (n = 46)	Dialysis group (n = 23)	KT group (n = 23)	p-value
MIP				
Observed (cm/H_2_O), median (IQR)	80 (60–100)	80 (70–100)	60 (60–100)	0.201
Predicted (cm/H_2_O), median (IQR)	75 (60–60)	90 (60–90)	90 (60–100)	0.498
Observed vs. predicted, p-value	0.977	0.306	0.215	
Patients reaching the predicted value, n (%)	20 (43.5)	12 (52.2)	8 (34.7)	0.239
MIP (%)^#^	88 ± 17	94 ± 12	82 ± 19	0.019
MEP				
Observed (cm/H_2_O), median (IQR)	70 (57.5–80.0)	60 (50–70)	70 (60–100)	0.020
Predicted (cm/H_2_O), median (IQR)	105 (90–120)	110 (85–115)	100 (90–130)	0.075
Observed vs. predicted, p-value	<0.001	<0.001	<0.001	
Patients reaching the predicted value, n (%)	2 (4.3)	0 (0)	2 (8.7)	0.155
MIP (%)^#^	66 ± 17	62 ± 12	71 ± 20	0.067
FEV1				
Observed (L), median (IQR)	2.1 (1.6–2.6)	2.0 (1.4–2.4)	2.2 (1.6–2.7)	0.328
Predicted (L), median (IQR)	3.6 (2.7–4.5)	4.4 (2.8–4.6)	2.9 (2.7–4.4)	0.126
Observed vs. Predicted, p-value	<0.001	<0.001	<0.001	
Patients reaching the predicted value, n (%)	0 (0)	0 (0)	0 (0)	NA
FEV1 (%)^#^	60 ± 18	55 ± 16	65 ± 19	0.091
FVC				
Observed (L), median (IQR)	2.7 (2.3–3.4)	2.5 (2.1–3.6)	2.8 (2.3–3.6)	0.166
Predicted (L), median (IQR)	3.7 (3.6–3.8)	3.6 (3.5–3.8)	3.7 (3.6–3.8)	0.472
Observed vs. Predicted, p-value	< 0.001	<0.001	<0.001	
Patients reaching the predicted value, n (%)	8 (17.4)	3 (13.0)	8 (21.8)	0.448
FVC (%)^#^	76 ± 22	72 ± 19	81 ± 24	0.159
Tiffeneau index (FEV1/FVC, %), median (IQR)	0.8 (0.6–0.9)	0.8 (0.7–0.9)	0.8 (0.6–0.9)	0.589

Abbreviations – MIP: maximal inspiratory pressure; MEP: maximal expiratory pressure; FEV1: forced expiratory volume in the first second; FVC: forced vital capacity; KT: kidney transplant.

Note – ^#^Scale variable obtained by dividing the observed by the predicted value x 100.

Both dialysis and KT patients performed worse than predicted in terms of FEV1 and FVC values. However, no differences were found between the groups regarding the observed values, the percentage of patients who achieved the predicted values, or the observed/predicted FEV1 and FVC ratios. Importantly, no patient achieved the predicted FEV1. The Tiffeneau index was similar between the groups (Table 2).

**Figure 1:**
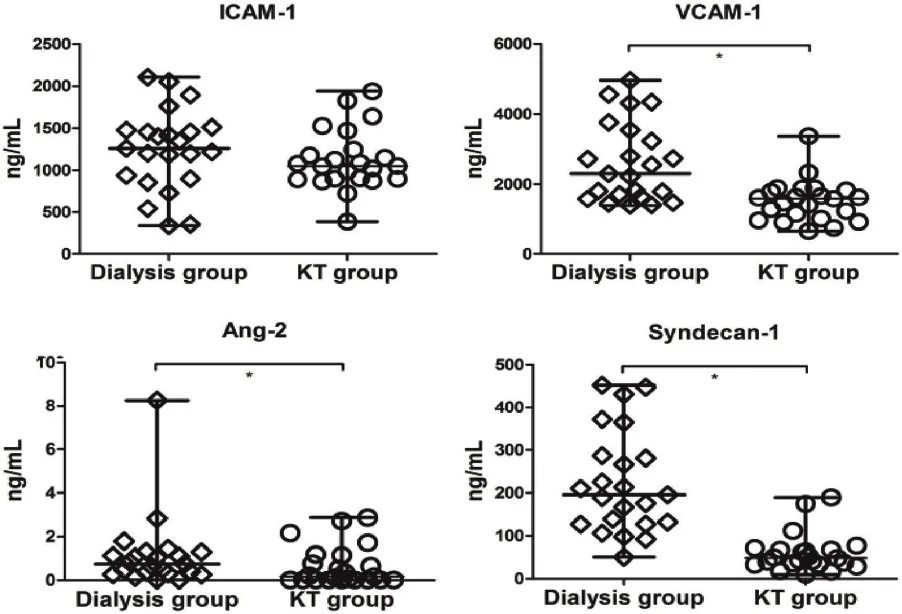
Biomarkers of endothelial damage in dialysis patients and kidney transplant recipients.

Compared with the dialysis patients, regarding biomarkers of endothelial damage, the KT group showed decreased levels of VCAM-1 (1,589 [IQR 1,009–1,827] vs. 2,302 [IQR 1,642–3,540] ng/mL; p = 0.001), angiopoietin-2 (0.17 [IQR 0.01–1.14] vs. 0.75 [IQR 0.30–1.29] ng/mL; p = 0.040), and syndecan-1 (47.9 [IQR 33.1–67.8] vs. 195.8 [IQR 126.9–286.8] ng/mL; p < 0.001). No statistical significance was observed in ICAM-1 levels (Figure 1).

Abbreviations – ICAM-1, intercellular adhesion molecule-1; VCAM-1, vascular cell adhesion molecule-1; ANG-2, angiopoietin-2.

Regarding the correlation between endothelial damage and pulmonary function, in the dialysis group, except for the inverse correlation between Ang-2 and FEV1 (rho = -0.435; p = 0.038), no other associations were observed between the biomarkers of endothelial damage and pulmonary function parameters. Some correlations were observed in the KT group. VCAM-1 was inversely correlated with MIP (rho = -0.433; p = 0.039) and MEP (rho = -0.444; p = 0.034). The same inverse correlation was observed between Ang-2 and FVC (rho = -0.492; p = 0.017); between syndecan-1 and FEV1 (rho = -0.416; p = 0.049) and FVC (rho = -0.648; p = 0.001); and between ICAM-1 and MIP (rho = -0.566; p = 0.005) (Table 3).

**Table 3.** Correlation between pulmonary function and biomarkers of endothelial damage in dialysis patients and kidney transplant recipients.

	Angiopoietin-2			Syndecan-1			VCAM-1			ICAM-1	
	rho	p		rho	p		rho	p		rho	p
Dialysis group											
MIP	-0.137	0.533		-0.007	0.974		0.109	0.619		-0.221	0.311
MEP	-0.379	0.075		0.388	0.067		0.165	0.451		0.068	0.758
FEV1	-0.435	0.038		0.405	0.055		0.105	0.632		0.093	0.671
FVC	-0.270	0.213		0.384	0.071		0.232	0.288		-0.058	0.791
KT group											
MIP	-0.334	0.119		-0.324	0.131		-0.433	0.039		-0.566	0.005
MEP	-0.332	0.121		-0.318	0.139		-0.444	0.034		-0.337	0.116
FEV1	-0.366	0.086		-0.416	0.049		-0.382	0.072		0.033	0.881
FVC	-0.492	0.017		-0.648	0.001		0.012	0.957		0.132	0.550

Abbreviations – MIP: maximal inspiratory pressure; MEP: maximal expiratory pressure; FEV1: forced expiratory volume in the first second; FVC: forced vital capacity; VCAM-1: vascular cell adhesion molecule-1; ICAM-1: intercellular adhesion molecule-1; KT: kidney transplant; rho: Spearman’s correlation coefficient.

## DISCUSSION

Our data demonstrated that, although KT patients present better endothelial function than dialysis patients, there are no significant differences in pulmonary function between these patients, suggesting that the pathophysiology of pulmonary dysfunction is multifactorial and complex and that some damage is probably not completely reversed by transplantation.

Chronic kidney disease is associated with physiological dysfunctions that negatively affect respiratory muscle strength and pulmonary function. Beyond uremic myopathy, bone demineralization, anemia, malnutrition, endothelial damage, oxidative damage, inflammation, and muscle disuse resulting from physical inactivity are also involved in pulmonary dysfunction^14,15,16,17^.

Kidney transplantation potentially reverses or mitigates these insults, restoring respiratory muscle strength and pulmonary function. However, evidence regarding this improvement in pulmonary function after KT is lacking. A study published in 2016 did not observe an increase in muscle strength at 30 days after KT. It is important to highlight that the effects of the early postoperative period may have interfered with the results^2^. Similarly, another study observed a decrease in respiratory muscle strength and pulmonary function after KT surgery^18^. Interestingly, these individuals did not reach the predicted values for these parameters, corroborating a study published in 2020, in which patients with kidney problems still presented muscle weakness when compared with the predicted values^4^. These findings reinforce the possible need for an adequate physiotherapy intervention and regular physical activity programs^4,18^.

The available evidence also failed to demonstrate complete recovery of pulmonary function when patients were evaluated late after transplantation. A 2006 study also found decreased respiratory muscle strength in patients with KT. The authors speculated that long-term immunosuppressive therapy, including the use of corticosteroids, and physical inactivity could explain the reduced MIP and MEP values and the lack of improvement after KT^1^.

It is important to highlight that the functional impairment extends beyond respiratory muscle strength in both groups. Limitations in lung volume and capacity in CKD patients are widely documented in the literature^19,20^. Although the mean observed-to-predicted FVC ratio among KT patients was above 80%, only 21.8% achieved the predicted values. The findings for FEV1 are even more pronounced, with low mean FEV1% results and no patient reaching the predicted values.

Endothelial dysfunction associated with CKD is widely known^21,22^. Inflammation and endothelial damage lead to changes in cell metabolism, causing a decrease in muscle functional capacity, resulting in impaired respiratory and peripheral muscle strength and resistance^14,23^. Our results suggest that KT is associated with less endothelial injury and that endothelial damage is inversely correlated with pulmonary function in this population. Despite the better endothelial function, transplanted patients maintain low performance in respiratory muscle strength and pulmonary function tests. Further longitudinal studies are needed to investigate the causality between these biomarkers and others, such as interleukins and FGF-23, and pulmonary function. Additionally, functional assessment variables, such as the six-minute walk test, should be evaluated.

Furthermore, the potential impact of the immunosuppressive therapy employed after kidney transplantation on respiratory muscle function cannot be disregarded and may, at least in part, have influenced the observed results regarding inspiratory muscle strength^24^.

Our study is the first to evaluate respiratory muscle strength and pulmonary function in ESKD patients, comparing hemodialysis and KT patients, and correlating these parameters with endothelial damage biomarkers. The analyses considering the predicted values according to age, sex, and height were valuable for a better interpretation of the data. However, some limitations should be pointed out. Despite matching, dialysis patients were older and had a higher prevalence of diabetes. Even in the presence of this imbalance, KT recipients did not perform better in pulmonary function tests, which reinforces our findings. There is no information on the CKD-mineral and bone disorder (CKD-MBD) profile or steroid doses, factors that might interfere with pulmonary function in KT recipients. However, patients had excellent renal function and a long time after KT, suggesting that they were clinically stable. Finally, despite the sample size calculation being based on FEV1 and FVC values, it might have been inadequate to show differences in respiratory muscle strength parameters, MIP and MEP.

## CONCLUSION

Despite improved endothelial function, long-term KT patients with adequate and stable renal function do not present improved pulmonary function when compared to dialysis patients, suggesting that recovery of renal function is not enough to reverse the long-term damage to respiratory muscle strength and pulmonary function secondary to CKD.

## Data Availability

The data collected and analyzed in this study are not publicly available due to ethical, legal, or privacy restrictions. However, they are available from the corresponding author upon reasonable request.
