# Supplementary material for: Pulmonary function and endothelial damage in patients undergoing renal replacement therapy: a cross-sectional study
Source: J Bras Nefrol. 2026 Aug 24;48(4):e20250222. doi: 10.1590/2175-8239-JBN-2025-0222en (PMC13502951; doi:10.1590/2175-8239-JBN-2025-0222en)
Supplement: Translation

# Translation: Funcionalidade pulmonar e danos endoteliais de pacientes em terapias de substituição renal: estudo transversal

**DOI:** 10.1590/2175-8239-JBN-2025-0222pt

**Authors:** Italo Caldas Silva, Gdayllon Cavalcante Meneses, Alice Maria Costa Martins, Alexandre Braga Libório, Elizabeth De Francesco Daher, Ronaldo de Matos Esmeraldo, Nataly Gurgel Campos, Tainá Veras de Sandes-Freitas

**Affiliations:** 1 Universidade Federal do Ceará, Programa de Pós-graduação em Ciências Médicas, Fortaleza, CE, Brasil.; 2 Universidade Federal do Ceará, Faculdade de Farmácia, Odontologia e Enfermagem, Fortaleza, CE, Brasil.; 3 Universidade de Fortaleza, Programa de Pós-graduação em Ciências Médicas, Fortaleza, CE, Brasil.; 4 Hospital Geral de Fortaleza, Setor de Transplante Renal, Fortaleza, Brasil.; 5 Universidade Federal do Ceará, Programa de Pós-graduação em Fisioterapia e Funcionalidade, Fortaleza, CE, Brasil.

**Keywords:** Transplante de Rim, Insuficiência Renal Crônica, Sistema Respiratório, Testes de Função Respiratória, Biomarcadores

## Abstract

**Objetivo:**

Comparar funcionalidade pulmonar e biomarcadores de lesão endotelial entre pacientes em hemodiálise com doença renal em estágio terminal (DRET) e receptores de transplante renal (TR).

**Métodos:**

Estudo transversal incluindo 23 pacientes em diálise por ≥24 meses e 23 pacientes transplantados há ≥ 12 meses, com taxa de filtração glomerular ≥ 40 mL/min/1,73 m^2^, pareados por sexo e idade. A funcionalidade pulmonar foi analisada por meio da pressão inspiratória e expiratória máxima (PIM e PEM), capacidade vital forçada (CVF), volume expiratório forçado no primeiro segundo (VEF1) e índice de Tiffeneau. O dano endotelial foi avaliado utilizando sindecano-1, molécula de adesão intercelular-1 (ICAM-1), molécula de adesão celular vascular (VCAM-1) e angiopoietina-2 (Ang-2).

**Resultados:**

Ambos os grupos apresentaram baixo desempenho nos testes de funcionalidade pulmonar. A porcentagem de pacientes que atingiram os valores previstos de PIM, PEM, VEF1 e CVF foi baixa e semelhante entre os grupos (43,5%, 4,3%, 0% e 17,4%, respectivamente). Não houve diferenças nas razões observado/previsto de PEM (66 ± 17%), VEF1 (60 ± 18%) e CVF (76 ± 22%), nem no índice de Tiffeneau (0,8 [IIQ 0,6–0,9]). Os pacientes submetidos a transplante renal apresentaram menor porcentagem de PIM (82 ± 19% vs. 94 ± 12%; p = 0,019). No grupo TR, o dano endotelial apresentou correlação inversa significativa com os parâmetros de funcionalidade pulmonar, e esse grupo apresentou níveis mais baixos de VCAM-1 (1.589 [IIQ 1.009–1.827] vs 2.302 [IIQ 1.642–3.540] ng/mL; p = 0,001), Ang-2 (0,17 [IIQ 0,01–1,14] vs 0,75 [IIQ 0,30–1,29] ng/mL; p = 0,040) e sindecano-1 (47,9 [IIQ 33,1–67,8] vs 195,8 [IIQ 126,9–286,8] ng/mL; p < 0,001).

**Conclusão:**

Apesar da melhor função endotelial, o transplante renal não esteve associado a uma funcionalidade pulmonar superior, sugerindo uma fisiopatologia multifatorial para o comprometimento pulmonar.

## INTRODUÇÃO

Pacientes com doença renal crônica (DRC) podem apresentar disfunção pulmonar secundária à anemia, hipervolemia, congestão pulmonar e, principalmente, ao comprometimento da musculatura respiratória causado por atrofia por desuso, desequilíbrio proteico, miopatia urêmica, inflamação crônica, estresse oxidativo e lesão endotelial^1,2,3,4^. O transplante renal (TR) potencialmente restaura a função renal e, consequentemente, a maioria desses distúrbios, melhorando a sobrevida a longo prazo e a qualidade de vida dos pacientes^5–8^.

Estudos anteriores apontaram para uma melhora, ainda que parcial, na funcionalidade pulmonar após o TR, atribuindo essa recuperação à reversão do ambiente urêmico^9,10^. Além da uremia, evidências indicam que o TR melhora a disfunção endotelial e reduz a inflamação sistêmica, o que também pode contribuir para a reversão parcial da disfunção pulmonar^11,12^.

É intuitivo supor que a recuperação da função renal após o TR resulte na restauração completa da função pulmonar. No entanto, há poucas evidências a esse respeito. Este estudo inovador visa comparar pacientes com doença renal em estágio terminal (DRET) em diálise com receptores de transplante renal estáveis. Para explorar ainda mais os mecanismos subjacentes ao comprometimento do sistema respiratório, avaliamos biomarcadores de disfunção endotelial em ambos os grupos.

## MÉTODOS

Estudo transversal incluindo pacientes com DRET em hemodiálise em um único centro de diálise e receptores de TR de um único centro de transplante, pareados 1:1 por idade e sexo.

Pacientes submetidos à hemodiálise por mais de 24 meses foram selecionados de forma não probabilística para o grupo de diálise. Para o grupo de TR, incluímos receptores de TR com ≥ 12 meses de acompanhamento, com idade entre 18 e 70 anos, com taxa de filtração glomerular (TFG) ≥ 40 mL/min/1,73 m^2^ estimada pela equação CKD-EPI, e que eram capazes de compreender e realizar os procedimentos de avaliação pulmonar. Pacientes com histórico de doença pulmonar obstrutiva crônica, asma, infarto agudo do miocárdio nos últimos três meses, doença cardíaca descompensada ou processo infeccioso ativo foram excluídos de ambos os grupos. Aqueles que haviam participado de qualquer estudo envolvendo exercício físico por um período inferior a seis meses ou que eram atletas ou praticantes regulares de exercícios (mais de duas vezes por semana) também foram excluídos. Para estimar o tamanho da amostra, foram considerados os seguintes fatores: a) nível de significância de 5%; b) poder estatístico de 90%; c) volume expiratório forçado no primeiro segundo (VEF1) e capacidade vital forçada (CVF) como variáveis de desfecho–considerando-se, com base na literatura^12,13^, uma diferença esperada de 0,86 e desvio padrão de 0,7 para o VEF1, e uma diferença esperada de 0,78 e desvio padrão de 0,7 para a CVF; d) estimativa de 20 a 30% de desistências, dados faltantes e perdas de seguimento.

O estudo foi conduzido de acordo com os padrões éticos da Resolução nº 466/2012 do Conselho Nacional de Saúde e da Declaração de Helsinque e aprovado pelo Comitê de Ética em Pesquisa (CEP) do Hospital Geral de Fortaleza (parecer nº 2.794.399). Cada paciente incluído no estudo assinou um Termo de Consentimento Livre e Esclarecido. Os registros e as informações individuais foram desidentificados e anonimizados antes da análise.

Quanto aos procedimentos do estudo, foram analisados os seguintes dados clínico-demográficos: sexo, idade, peso, altura, índice de massa corporal (IMC), histórico de diabetes, hipertensão, tabagismo prévio ou atual, causas da DRC em estágio terminal, tempo de diálise e tempo após o TR.

Os parâmetros de funcionalidade pulmonar analisados foram: pressão expiratória máxima (PEM), pressão inspiratória máxima (PIM), VEF1, CVF e índice de Tiffeneau. No grupo de diálise, as avaliações foram realizadas antes das sessões de tratamento. As medidas de PIM e PEM foram realizadas utilizando um manovacuômetro MR^®^. Após três medições, o maior valor foi considerado para análise. Um espirômetro portátil ONE FLOW RANGE (*Clement Clarke International*) foi utilizado para avaliar os parâmetros espirométricos (VEF1 e CVF), de acordo com os critérios estabelecidos pela *American Thoracic Society* (1995). Para estimar os valores previstos de PIM e PEM, foram empregadas as seguintes fórmulas^14^:

- PIM prevista = 149,33 - 1,14 × idade, se masculino; e 74,25 - 0,46 × idade, se feminino.
- PEM prevista = 183,31 - 1,26 × idade, se masculino; e 119,35 - 0,68 × idade, se feminino.

Para estimar os valores previstos de VEF1 e CVF, foram empregadas as seguintes fórmulas^12^:

- VEF1 previsto = (altura × 0,0473) - (idade × 0,0281) - 3,145, se masculino; e (altura × 0,0338) - (idade × 0,0210) - 1,782, se feminino.
- CVF prevista = 4,569 - (altura × 0,0590) - (idade × 0,0281), se masculino; e 2,967 - (altura × 0,0433) - (idade × 0,0164), se feminino.

Os biomarcadores endoteliais foram quantificados a partir de amostras de soro isoladas, utilizando kits ELISA humanos.

Os ensaios ELISA foram realizados de acordo com as instruções de uso do fabricante. Sindecano-1: kit ab46506 (Abcam^®^); molécula de adesão intercelular-1 (ICAM-1): kit ab47349 (Abcam^®^); molécula de adesão celular vascular (VCAM-1): kit ab47355 (Abcam^®^); angiopoietina-2 (Ang-2): kit DY623 (R&D Systems^®^). As amostras de sangue para análise de biomarcadores foram coletadas 10 a 30 minutos antes da sessão de hemodiálise em pacientes em diálise. No grupo de transplante renal (TR), o material sanguíneo foi coletado antes do teste de função pulmonar. As amostras foram coletadas em tubos apropriados para isolamento de soro e, em seguida, o soro foi aliquotado e armazenado adequadamente a -80 °C até a realização das análises.

A análise estatística foi realizada. Variáveis contínuas com distribuição normal foram resumidas como média ± desvio padrão e comparadas utilizando o teste t de Student. Variáveis com distribuição não normal foram expressas como mediana e intervalo interquartil (IIQ) e comparadas utilizando o teste de Mann-Whitney. O teste de Shapiro-Wilk foi empregado para verificar a normalidade da distribuição. Variáveis categóricas foram resumidas como frequências e proporções e comparadas utilizando os testes exato de Fisher ou do qui-quadrado (*χ* ^2^). O coeficiente de correlação de Spearman (rho) foi utilizado para avaliar as correlações entre variáveis numéricas com distribuição normal e não normal. Os dados foram analisados utilizando o pacote estatístico SPSS versão 23.0 para Macintosh (IBM, Armonk, NY, EUA). Os gráficos de dispersão vertical foram criados utilizando o GraphPad Prism 5 para Windows (versão 5.01). Valores de p < 0,05 foram considerados estatisticamente significativos.

## RESULTADOS

Um total de 46 pacientes foi incluído no estudo, 23 em cada grupo. Apesar da compatibilidade, os pacientes do grupo de diálise eram mais velhos do que os do grupo de transplante renal (51,1 ± 5,7 vs. 46,0 ± 5,0 anos; p = 0,002). Os grupos diferiram quanto à etiologia da DRC e ao histórico de diabetes, com uma proporção maior de pacientes diabéticos entre os que estavam em diálise (47,9% vs. 4,3%; p = 0,001). A Tabela 1 apresenta os dados demográficos.

**Tabela 1.** Características clínicas e demográficas de pacientes dialíticos e transplantados renais.

	Total (N = 46)	Grupo diálise (N = 23)	Grupo TR (N = 23)	Valor de p
Sexo masculino, n (%)	23 (50)	12 (52,2)	11 (47,9)	0,774
Idade, média ± desvio padrão	49,5 ± 5,9	51,1 ± 5,7	46,0 ± 5,0	0,002
IMC (kg/m^2^), média ± desvio padrão	25,6 ± 3,1	26,5 ± 3,5	24,7 ± 2,5	0,058
Tempo de diálise (meses), mediana	36,0 (21,8–49,5)	36,0 (21,5–48,0)	36,0 (24,0–54,0)	0,851
Tempo pós-TR (meses), mediana	24,0 (17,5–48,0)	NA	24 (17,5–48,0)	NA
Etiologia da DRC, n (%)				0,001
GNC	16 (34,8)	8 (34,8)	8 (34,8)	
Hipertensão	10 (21,7)	7 (30,4)	3 (13,0)	
Desconhecido	10 (21,7)	0 (0)	10 (43,5)	
Diabetes	9 (19,5)	8 (34,8)	1 (8,7)	
DRP	1 (2,2)	0 (0)	1 (4,3)	
Comorbidades, n (%)				
Hipertensão	28 (60,9)	16 (69,6)	12 (52,2)	0,232
Diabetes	12 (26,1)	11 (47,9)	1 (4,3)	0,001
Fumante atual ou prévio, n (%)	8 (17,4)	06 (26,1)	2 (8,7)	0,124
TFGe (mL/min/1,73 m^2^), média ± desvio padrão	75,2 ± 27,6	NA	75,2 ± 27,6	NA
Regime de imunossupressores, n (%)				NA
TAC-mTORi	15 (36,6)	NA	15 (65,2)	
TAC-MPA	9 (19,6)	NA	9 (39,1)	
Regime com corticosteroides, n (%)	9 (19,6)	NA	9 (39,1)	NA

Abreviaturas – TR: transplante renal; IMC: índice de massa corporal; DRC: doença renal crônica; GNC: glomerulonefrite crônica; DRP: doença renal policística; TFGe: taxa de filtração glomerular estimada; TAC: tacrolimus; mTORi: inibidor da mTOR; MPA: micofenolato.

Em relação aos parâmetros da função pulmonar, não houve diferenças entre os grupos quanto à PIM observada ou à porcentagem de pacientes que atingiram a PIM prevista. Paradoxalmente, a razão PIM observada/prevista foi menor entre os receptores de transplante (82 ± 19% vs. 94 ± 12%; p = 0,019). Nos grupos de transplante renal e diálise, os valores de PIM observados não foram significativamente menores do que os valores previstos. Em relação à PEM, os pacientes transplantados apresentaram valores observados mais altos (70 [IIQ 60–100] vs. 60 [IIQ 50–70] cm/H^2^O; p = 0,020), embora não tenham sido constatadas diferenças significativas na porcentagem de pacientes que atingiram a PEM prevista ou na razão PEM observada/prevista. Ambos os grupos apresentaram valores de PEM menores do que os previstos para idade, altura e sexo (Tabela 2).

**Tabela 2.** Funcionalidade pulmonar em pacientes dialíticos e transplantados renais.

	Total (n = 46)	Grupo diálise (n = 23)	Grupo TR (n = 23)	Valor de p
PIM				
Observado (cm/H_2_O), mediana (IIQ)	80 (60–100)	80 (70–100)	60 (60–100)	0,201
Previsto (cm/H_2_O), mediana (IIQ)	75 (60–60)	90 (60–90)	90 (60–100)	0,498
Observado vs. Previsto, valor de p	0,977	0,306	0,215	
Pacientes que atingiram o previsto, n (%)	20 (43,5)	12 (52,2)	8 (34,7)	0,239
PIM (%)^#^	88 ± 17	94 ± 12	82 ± 19	0,019
PEM				
Observado (cm/H_2_O), mediana (IIQ)	70 (57,5–80,0)	60 (50–70)	70 (60–100)	0,020
Previsto (cm/H_2_O), mediana (IIQ)	105 (90–120)	110 (85–115)	100 (90–130)	0,075
Observado vs. Previsto, valor de p	<0,001	<0,001	<0,001	
Pacientes que atingiram o previsto, n (%)	2 (4,3)	0 (0)	2 (8,7)	0,155
PEM (%)^#^	66 ± 17	62 ± 12	71 ± 20	0,067
VEF1				
Observado (L), mediana (IIQ)	2,1 (1,6–2,6)	2,0 (1,4–2,4)	2,2 (1,6–2,7)	0,328
Previsto (L), mediana (IIQ)	3,6 (2,7–4,5)	4,4 (2,8–4,6)	2,9 (2,7–4,4)	0,126
Observado vs. Previsto, valor de p	<0,001	<0,001	<0,001	
Pacientes que atingiram o previsto, n (%)	0 (0)	0 (0)	0 (0)	NA
VEF1 (%)^#^	60 ± 18	55 ± 16	65 ± 19	0,091
CVF				
Observado (L), mediana (IIQ)	2,7 (2,3–3,4)	2,5 (2,1–3,6)	2,8 (2,3–3,6)	0,166
Previsto (L), mediana (IIQ)	3,7 (3,6–3,8)	3,6 (3,5–3,8)	3,7 (3,6–3,8)	0,472
Observado vs. Previsto, valor de p	< 0,001	<0,001	<0,001	
Pacientes que atingiram o previsto, n (%)	8 (17,4)	3 (13,0)	8 (21,8)	0,448
CVF (%)^#^	76 ± 22	72 ± 19	81 ± 24	0,159
Índice de Tiffeneau (VEF1/CVF, %), mediana (IIQ)	0,8 (0,6–0,9)	0,8 (0,7–0,9)	0,8 (0,6–0,9)	0,589

Abreviaturas – PIM: pressão inspiratória máxima; PEM: pressão expiratória máxima; VEF1: volume expiratório forçado no primeiro segundo; CVF: capacidade vital forçada; TR: transplante renal.

Nota – ^#^Variável de escala obtida pela divisão do valor observado pelo valor previsto × 100.

Tanto os pacientes em diálise quanto os receptores de transplante renal apresentaram desempenho pior do que o previsto nos valores de VEF1 e CVF. No entanto, não foram encontradas diferenças entre os grupos quanto aos valores observados, à porcentagem de pacientes que atingiram os valores previstos ou às razões VEF1 observado/previsto e CVF observado/previsto. É importante ressaltar que nenhum paciente atingiu o VEF1 previsto. O índice de Tiffeneau foi semelhante entre os grupos (Tabela 2).

Em comparação com os pacientes em diálise, no que diz respeito aos biomarcadores de dano endotelial, o grupo TR apresentou níveis reduzidos de VCAM-1 (1.589 [IIQ 1.009–1.827] vs. 2.302 [IIQ 1.642–3.540] ng/mL; p = 0,001), angiopoietina-2 (0,17 [IIQ 0,01–1,14] vs. 0,75 [IIQ 0,30–1,29] ng/mL; p = 0,040) e sindecano-1 (47,9 [IIQ 33,1–67,8] vs. 195,8 [IIQ 126,9–286,8] ng/mL; p < 0,001). Não foi observada significância estatística nos níveis de ICAM-1 (Figura 1).

**Figure 1:** Biomarcadores de lesão endotelial em pacientes dialíticos e transplantados renais.

Abreviaturas – ICAM-1, molécula de adesão intercelular 1; VCAM-1, molécula de adesão celular vascular 1; ANG-2, angiopoietina-2.

Quanto à correlação entre dano endotelial e função pulmonar, no grupo de diálise, com exceção da correlação inversa entre Ang-2 e VEF1 (rho = -0,435; p = 0,038), nenhuma outra associação foi observada entre biomarcadores de dano endotelial e parâmetros de função pulmonar. Algumas correlações foram observadas no grupo de transplante renal. VCAM-1 apresentou correlação inversa com PIM (rho = -0,433; p = 0,039) e PEM (rho = -0,444; p = 0,034). A mesma correlação inversa foi observada entre Ang-2 e CVF (rho = -0,492; p = 0,017); entre sindecano-1 e VEF1 (rho = -0,416; p = 0,049) e CVF (rho = -0,648; p = 0,001); e entre ICAM-1 e PIM (rho = -0,566; p = 0,005) (Tabela 3).

**Tabela 3.** Correlação entre funcionalidade pulmonar e biomarcadores de dano endotelial em pacientes dialíticos e transplantados renais.

	Angiopoietina-2			Sindecano-1			VCAM-1			ICAM-1	
	rho	p		rho	p		rho	p		rho	p
Grupo diálise											
PIM	-0,137	0,533		-0,007	0,974		0,109	0,619		-0,221	0,311
PEM	-0,379	0,075		0,388	0,067		0,165	0,451		0,068	0,758
VEF1	-0,435	0,038		0,405	0,055		0,105	0,632		0,093	0,671
CVF	-0,270	0,213		0,384	0,071		0,232	0,288		-0,058	0,791
Grupo TR											
PIM	-0,334	0,119		-0,324	0,131		-0,433	0,039		-0,566	0,005
PEM	-0,332	0,121		-0,318	0,139		-0,444	0,034		-0,337	0,116
VEF1	-0,366	0,086		-0,416	0,049		-0,382	0,072		0,033	0,881
CVF	-0,492	0,017		-0,648	0,001		0,012	0,957		0,132	0,550

Abreviaturas – PIM: pressão inspiratória máxima; PEM: pressão expiratória máxima; VEF1: volume expiratório forçado no primeiro segundo; CVF: capacidade vital forçada; VCAM-1: molécula de adesão celular vascular 1; ICAM-1: molécula de adesão intercelular 1; rho: coeficiente de correlação de Spearman.

## DISCUSSÃO

Nossos dados demonstraram que, embora os pacientes transplantados renais apresentem melhor função endotelial do que os pacientes em diálise, não há diferenças significativas na funcionalidade pulmonar entre esses pacientes, sugerindo que a fisiopatologia da disfunção pulmonar é multifatorial e complexa e que, provavelmente, alguns danos não são completamente revertidos pelo transplante.

A doença renal crônica está associada a disfunções fisiológicas que afetam negativamente a força muscular respiratória e a função pulmonar. Além da miopatia urêmica, a desmineralização óssea, a anemia, a desnutrição, o dano endotelial, o dano oxidativo, a inflamação e o desuso muscular decorrente da inatividade física também estão envolvidos na disfunção pulmonar^14–17^.

O transplante renal potencialmente reverte ou atenua esses danos, restaurando a força e a função pulmonar. No entanto, faltam evidências sobre essa melhora na funcionalidade pulmonar após o transplante renal. Um estudo publicado em 2016 não observou aumento da força muscular 30 dias após o transplante renal. É importante destacar que os efeitos do período pós-operatório inicial podem ter interferido nos resultados^2^. De forma semelhante, outro estudo observou uma diminuição da força muscular respiratória e da função pulmonar após a cirurgia de TR^18^. Curiosamente, esses indivíduos não atingiram os valores previstos desses parâmetros, corroborando um estudo publicado em 2020, no qual pacientes com problemas renais ainda apresentavam fraqueza muscular quando comparados aos valores previstos^4^. Esses achados reforçam uma possível necessidade de implementar intervenção fisioterapêutica adequada e programas regulares de atividade física^4,18^.

As evidências disponíveis também não demonstraram recuperação completa da funcionalidade pulmonar quando os pacientes foram avaliados tardiamente após o transplante. Um estudo de 2006 também encontrou diminuição da força muscular respiratória em pacientes com TR. Os autores especularam que a terapia imunossupressora de longo prazo, como a terapia com corticosteroides, e a inatividade física poderiam explicar os valores reduzidos de PIM e PEM e a falta de melhora após o TR^1^.

É importante destacar que o comprometimento funcional vai além da força muscular respiratória em ambos os grupos. As limitações de volume e capacidade pulmonar em pacientes com DRC são amplamente documentadas na literatura^19,20^. Embora a média dos pacientes transplantados renais apresentasse uma relação CVF observada/prevista acima de 80%, apenas 21,8% atingiram os valores previstos. Os resultados relacionados ao VEF1 são ainda mais expressivos, com baixas médias de VEF1% e nenhum paciente atingindo os valores previstos.

A disfunção endotelial associada à DRC é amplamente conhecida^21,22^. A inflamação e o dano endotelial levam a alterações no metabolismo celular, causando uma diminuição na capacidade funcional muscular, resultando em comprometimento da força e da resistência muscular respiratória e periférica^14,23^. Nossos resultados sugerem que o transplante renal está associado a menor lesão endotelial e que o dano endotelial está inversamente correlacionado com a funcionalidade pulmonar nessa população. Apesar da melhor função endotelial, os pacientes transplantados mantêm baixo desempenho nos testes de força e função pulmonar. São necessários mais estudos longitudinais que abordem a relação de causalidade entre esses biomarcadores e outros, como as interleucinas e o FGF-23, e a funcionalidade pulmonar. Além disso, variáveis de avaliação funcional, como o teste da caminhada de seis minutos, devem ser analisadas.

Vale ressaltar que não se pode descartar a influência do regime imunossupressor utilizado após o TR sobre a função da musculatura respiratória, o que pode ter contribuído, em parte, para os resultados observados na força muscular inspiratória^24^.

Nosso estudo é pioneiro na avaliação da força muscular respiratória e da função pulmonar em pacientes com DRC em estágio terminal, comparando pacientes em hemodiálise e transplantados renais, e correlacionando esses parâmetros com biomarcadores de dano endotelial. As análises que consideraram os valores previstos de acordo com idade, sexo e altura foram valiosas para uma melhor interpretação dos dados. No entanto, algumas limitações devem ser apontadas. Apesar do pareamento, os pacientes em diálise eram mais velhos e apresentavam maior prevalência de diabetes. Mesmo diante desse desequilíbrio, os receptores de transplante renal não apresentaram melhor desempenho nos testes de funcionalidade pulmonar, o que reforça nossos achados. Não há informações sobre o perfil do distúrbio mineral e ósseo da DRC (DMO-DRC) e as doses de esteroides, fatores que podem interferir na funcionalidade pulmonar dos receptores de transplante renal. No entanto, os pacientes apresentavam excelente função renal e um longo período após o transplante, sugerindo que eram pacientes clinicamente estáveis. Por fim, apesar de o cálculo do tamanho da amostra ter se baseado nos valores de VEF1 e CVF, é possível que tenha sido inadequado para demonstrar diferenças nos parâmetros de força muscular, PIM e PEM.

## CONCLUSÃO

Apesar da melhora na função endotelial, os pacientes transplantados renais de longa duração, com função renal adequada e estável, não apresentam melhora na funcionalidade pulmonar quando comparados aos pacientes em diálise, sugerindo que a recuperação da função renal não é suficiente para reverter os danos de longo prazo na força muscular respiratória e na função pulmonar secundários à DRC.

## Data Availability

Os dados coletados e analisados neste estudo não estão disponíveis publicamente devido a restrições éticas, legais ou de privacidade. No entanto, podem ser obtidos mediante solicitação razoável ao autor correspondente.
